# Association of Industry and Academic Sponsorship With Negative Phase 3 Oncology Trials and Reported Outcomes on Participant Survival: A Pooled Analysis

**DOI:** 10.1001/jamanetworkopen.2019.3684

**Published:** 2019-05-10

**Authors:** Alfredo Addeo, Glen J. Weiss, Bishal Gyawali

**Affiliations:** 1 Department of Oncology, University Hospital of Geneva, Geneva, Switzerland; 2 Department of Medicine, Beth Israel Deaconess Medical Center, Harvard Medical School, Boston, Massachusetts; 3 Department of Medicine, Brigham and Women’s Hospital, Harvard Medical School, Boston, Massachusetts; 4 Division of Cancer Care and Epidemiology, Department of Oncology and Department of Public Health Sciences, Queen’s University, Kingston, Ontario, Canada

## Abstract

**Importance:**

Only 3.4% of cancer drugs evaluated in phase 1 trials are approved by the US Food and Drug Administration, with most failing in phase 3 trials.

**Objective:**

To investigate whether an association exists between the sponsorship and conduct of a negative phase 3 randomized clinical trial (RCT) investigating a cancer drug that lacked supporting phase 2 trial evidence for that drug, and to evaluate the association with overall survival among patients randomized to the experimental arm of such phase 3 trials.

**Data Sources:**

Articles in the *Lancet*, *Lancet Oncology*, *JAMA*, *JAMA Oncology*, and *Journal of Clinical Oncology* published between January 2016 and June 2018 were searched.

**Study Selection:**

Phase 3 RCTs of cancer drugs that failed to improve the primary end point were selected and any prior phase 2 trial of the same drug that supported the phase 3 trial was selected without any date or journal restrictions.

**Data Extraction and Synthesis:**

Percentages of negative phase 3 RCTs of cancer drugs that lacked any phase 2 evidence, had a negative phase 2 trial, or had a positive phase 2 study were extracted. Associations were assessed using the Fisher exact test. Pooled hazard ratios and 95% CIs for the overall survival of patients enrolled in these negative phase 3 RCTs were estimated using a random-effects model.

**Main Outcomes and Measures:**

Negative phase 3 RCTs with a lack of a phase 2 trial or the presence of a negative phase 2 trial and overall survival of enrolled patients in the phase 3 RCTs.

**Results:**

In this meta-epidemiological study, 67 negative phase 3 RCTs on cancer drugs, which included 64 600 patients, met the criteria of being sponsored by industry or academic groups, of which 42 RCTs (63%) were industry sponsored and the remaining 25 RCTs (37%) were academic. A phase 2 trial was not available for 28 of these trials (42%). Of 29 trials (43%) with a phase 2 trial available, 8 trials (28%) failed to meet their primary end points and 5 of those were industry sponsored. There was no association with overall survival for patients participating in these negative phase 3 RCTs (pooled hazard ratio, 0.99; 95% CI, 0.96-1.02). When the pooled analysis was limited to the 27 RCTs with a hazard ratio above 1.00, the overall pooled hazard ratio for overall survival was 1.11 (95% CI, 1.06-1.16). No association between having a negative or undefined phase 2 trial and trial sponsorship was found using the Fisher exact test.

**Conclusions and Relevance:**

More than 40% of the negative phase 3 RCTs in oncology published in these 5 journals were conducted without a supporting phase 2 trial and were sponsored by both academia and industry. Running such trials not only may risk loss of resources owing to a failed trial but also may be associated with decreased patient survival. Further research and regulations in this area appear warranted.

## Key Points

**Question:**

Does an association exist between the sponsorship and conduct of phase 3 randomized clinical trials for cancer drugs despite negative or absent phase 2 trials for the drug, and does an association exist for overall patient survival and such phase 3 trials?

**Findings:**

This analysis of 67 studies found that both industry and academia conducted negative phase 3 trials of cancer drugs. No association was found between trial sponsorship and lack of a positive phase 2 trial; there was no association with patient overall survival, although the survival hazard ratio was greater than 1 for 37% of such trials.

**Meaning:**

Phase 3 trials conducted without supporting phase 2 trial evidence risks loss of resources owing to trial failure and may be associated with decreased patient survival.

## Teaser

This study assesses whether an association exists between the sponsorship and conduct of a phase 3 randomized clinical trial investigating a cancer drug and the lack of supporting phase 2 trial evidence for that drug.

## Introduction

Phase 3 randomized clinical trials (RCTs) are the final barrier in establishing the efficacy of cancer drugs. One study estimates that only 3.4% of cancer drugs being evaluated in phase 1 trials are approved by the US Food and Drug Administration, with most (35.5%) failing in the phase 3 stage when all indications are considered.^1^ For lead indications only, the numbers look better but are still disappointing, with 11.4% probability of success overall and 48.5% probability of success at the phase 3 stage of development. Phase 3 RCTs usually involve more patients and resources than earlier phases and are costly undertakings. According to a 2014 report, the cost of a cancer drug trial was $22.1 million, $11.2 million, and $4.5 million, for a phase 3, phase 2, and phase 1 trial, respectively.^2^ Thus, a drug failing the phase 3 trial (negative phase 3 trial) is an undesirable outcome, even from an economic point of view.

However, the larger concern is the effect on human resources. First, patient resources for trial enrollment are scarce given that only 3% of patients with cancer in the United States participate in trials and that nearly 60% of phase 3 trials fail to achieve minimum patient enrollment.^3^ Second, a trial involves significant investment of time and logistics from health care professionals and patients. Third, a failed phase 3 trial also means unmet patient expectations. Thus, although negative RCTs can provide us with important knowledge for future research and patient care, precautions can be taken to minimize the frequency of negative RCTs, especially if they can be avoided. The US Food and Drug Administration resource “Information for Consumers (Drugs)” states that “if the phase 2 trials indicate that the drug may be effective—and the risks are considered acceptable, given the observed efficacy and the severity of the disease—the drug moves to phase 3.”^4^ Another document on the clinical review of investigational new drug applications also states that a phase 2 trial review is conducted on completion before initiation of phase 3 trials, during which questions such as whether sufficient data are available to plan a phase 3 program are asked.^5^ However, in a previous study, our group discovered that less than 20% of the negative phase 3 RCTs on cancer had a supportive phase 2 trial.^6^ One hypothesis for such a practice is that it is financially lucrative for the industry to continue to test compounds that have low probability of success in a phase 3 trial because even a chance finding of a positive outcome will offset all associated costs.^7^ However, associations of sponsors of such negative phase 3 RCTs with phase 2 trial outcomes or the association of such negative phase 3 RCTs on patient survival has not been studied systematically to our knowledge.

In the present study, using a larger data set of negative phase 3 RCTs, we investigated whether an association existed between the sponsorship of negative phase 3 RCTs and the lack of supportive prior phase 2 trial evidence. We also studied what proportion of the industry-sponsored negative phase 3 RCTs had a history of the experimental drug being acquired or licensed from a small pharmaceutical company (defined as having a market capitalization of less than $2 billion). We hypothesized that such an arrangement might motivate the sponsor to pursue a phase 3 RCT to recoup the costs of the investment. Finally, we investigated whether there was an association with overall survival (OS) among patients who were enrolled in these negative phase 3 RCTs.

## Methods

This study was conducted based on modified Preferred Reporting Items for Systematic Reviews and Meta-analyses (PRISMA) reporting guidelines for meta-epidemiological studies.^8^ We searched the top oncology journals based on their impact factor (*Lancet Oncology*, *JAMA Oncology*, and *Journal of Clinical Oncology*) and their parent journals (*Lancet* and *JAMA*) between January 2016 and June 2018 for any negative phase 3 RCTs of cancer drugs. We excluded the RCTs that did not involve a cancer drug in any arm, such as RCTs of radiotherapies or surgical procedures in both the arms. We extracted the hazard ratio (HR) and 95% CI for OS for each of the included trials, where available. We also categorized the sponsors of these negative phase 3 RCTs into academic (cooperative groups) or industry based on the posted ClinicalTrials.gov registration information. We then searched the literature and meeting abstracts for any prior phase 2 trial of the same drug that supported the phase 3 trial. We categorized these findings into yes, no, and not applicable (NA). Not applicable was applied to drugs that had already been approved for later lines of therapy or in an advanced setting but the phase 3 RCT tested the drug in an earlier or curative setting. For those trials categorized as yes for any prior phase 2 trial, we categorized the outcomes of the phase 2 trials into positive, negative, or inconclusive based on whether the primary end point of the phase 2 trial was met, unmet, or unstated, respectively. Finally, for industry-sponsored trials, to find out if the drug was purchased or licensed from a small pharmaceutical company (defined as having a market capitalization of less than $2 billion), we used search terms in Google (eg, the name of the small pharmaceutical company, the drug or compound name, and terms such as *purchase*, *acquired*, *bought*, *licensed*, *merger*, *sells*, *royalties*, or *option*) and then reviewed the news article or press release for details, if any, regarding a transaction or business arrangement or deal with another pharmaceutical company or entity. All study and data extractions as well as categorizations were made by 2 of us (A.A. and G.W.) and rechecked by the third author (B.G.). Any discrepancies were resolved by consensus among the 3 authors.

### Statistical Analysis

The associations of availability and of outcomes of phase 2 trials with sponsorship of negative phase 3 trials were assessed using the Fisher exact test. We also conducted a pooled analysis using a random-effects model to estimate the summary HR for OS across the negative trials. A random-effects model was used to account for the heterogeneity across the trials. All analyses were conducted in Stata, version 15 (StataCorp), and a 2-sided *P* < .05 was considered statistically significant.

## Results

We found 67 negative phase 3 RCTs,^9,10,11,12,13,14,15,16,17,18,19,20,21,22,23,24,25,26,27,28,29,30,31,32,33,34,35,36,37,38,39,40,41,42,43,44,45,46,47,48,49,50,51,52,53,54,55,56,57,58,59,60,61,62,63,64,65,66,67,68,69,70,71,72,73,74,75^ which included 64 600 patients, that met our criteria for inclusion, of which 42 RCTs (63%) were industry sponsored and the remaining 25 RCTs (37%) were academic (eTable in the Supplement).^9,10,11,12,13,14,15,16,17,18,19,20,21,22,23,24,25,26,27,28,29,30,31,32,33^ A phase 2 trial was not available for 28 (42%) of these negative phase 3 RCTs, it was available for 29 (43%) of them, and it was not applicable for 10 (15%) of them. Excluding the 10 nonapplicable trials, the availability of a phase 2 trial was not associated with negative phase 3 trial sponsorship (*P* > .99, assessed using the Fisher exact test). Of the 29 trials in which a phase 2 trial was available, 8 trials (28%; 14% of all 57 trials) failed to meet the primary end point, whereas the phase 2 trial was inconclusive in 5 cases (17%). The phase 2 trial met its primary end point and was considered positive in 16 trials (55%, or 28% of 57 trials). There was no association between having a negative or undefined phase 2 trial and phase 3 trial sponsorship (*P* > .99, assessed using the Fisher exact test).

Of the 8 RCTs that were conducted despite a negative phase 2 trial, 5 were industry sponsored. Two of the 3 academic phase 3 RCTs were conducted despite a negative phase 2 trial because the phase 2 trial showed beneficial effects in a subgroup of biomarker-positive patients. One was the Alliance CALGB 30801 trial of celecoxib in non–small cell lung cancer with cyclooxygenase-2 overexpression that was conducted despite a negative phase 2 trial because cyclooxygenase-2 overexpression was supposed to be a predictive biomarker.^9^ The other was the RCT of lapatinib in bladder cancer, which was also tested in a biomarker-selected population (ERBB1/2 [formerly HER1/2] positive) in phase 3, despite a negative phase 2 trial in an unselected population.^34^ Of the 5 industry-sponsored trials that were conducted despite a negative phase 2 trial, 4 trials were associated with purchase or licensure of the molecule from a small pharmaceutical company.

Of the 67 RCTs, 59 had OS HR and CI data available. When pooled using a random-effects model, the patients participating in these trials were not associated with poorer OS (pooled HR, 0.99; 95% CI, 0.96-1.02) with no subgroup differences by trial sponsorship or by phase 2 trial results. However, the HR was greater than 1.00 in 27 RCTs (46%). When the pooled analysis was limited to these 27 RCTs,^9,10,11,12,13,14,15,16,17,18,19,20,21,22,23,24,25,26,27,28,29,30,31,32,33,35,36^ the overall pooled HR for overall survival was 1.11 (95% CI, 1.06-1.16) (Figure).

**Figure.**
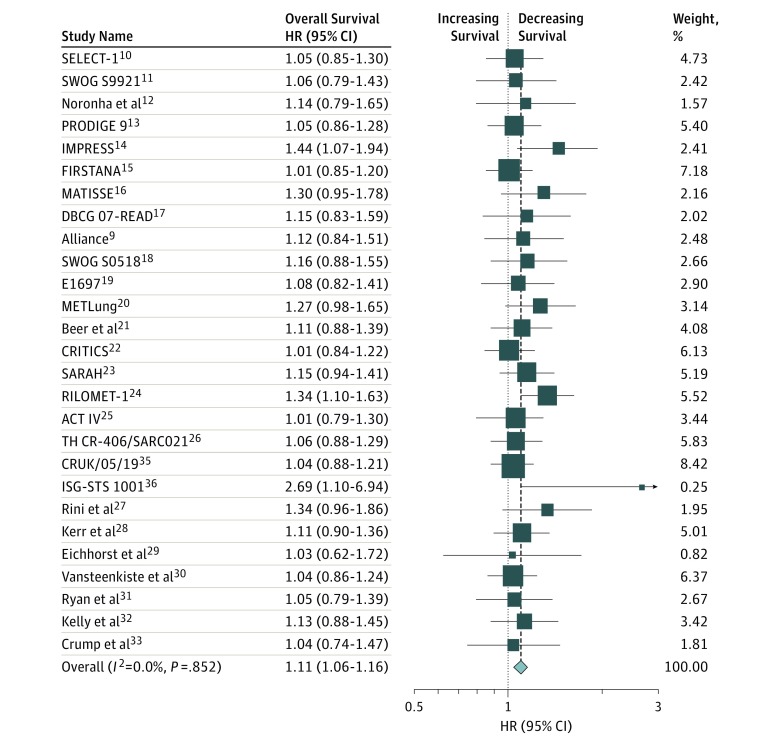
Forest Plot of Overall Survival Hazard Ratio (HRs) for the 27 Phase 3 Randomized Clinical Trials With an HR of More Than 1 Included in the Study The size of each box represents the weight by random-effects analysis of the contribution of each study to the weight of each sample in the meta-analysis. The vertical dashed line indicates the point of summary HR and the diamond indicates the 95% CI for the summary hazard ratio. Values less than 1 (and symbols to the left of HR = 1) reflect protective effects of treatment and values more than 1 (symbols to the right of HR = 1) reflect detrimental effects of treatment on survival.

## Discussion

Of the negative phase 3 RCTs of cancer drugs, 42% did not have phase 2 trial evidence and only 28% had a positive phase 2 trial. Nearly 14% of negative phase 3 RCTs were conducted despite a negative phase 2 trial. The availability of a phase 2 trial or whether or not the phase 2 trial was positive was not associated with trial sponsorship, but of the 5 industry-sponsored negative phase 3 RCTs, 4 were associated with drug purchase or licensure from a small pharmaceutical company. Patients who were enrolled in the negative phase 3 RCTs were not associated with poorer OS although when limited to 46% of negative phase 3 RCTs reporting an HR greater than 1.00, the pooled HR showed a significant increase in mortality among the participants randomized to the experimental arm of the trials.

There are several hypotheses as to why a cancer drug may be tested in a phase 3 RCT despite having no or even negative phase 2 trial evidence. Among industry-sponsored trials, we found that acquisition of the investigational drug from small pharmaceutical companies could be one factor. Financial milestones among the small pharmaceutical companies to move a compound from phase 2 to phase 3 trials can influence decisions. However, in the case of academia-sponsored trials, a belief in the biomarker-based subgroup analysis of phase 2 trials seemed to be a motivation for conducting a larger phase 3 trial in the biomarker-enriched subgroup population.

Negative phase 3 RCTs are not futile or meaningless; they are important to advance our understanding of disease and drugs. Nevertheless, because of the financial, human, logistic, and time resources needed to conduct a phase 3 RCT and the patients’ expectation of therapeutic benefit, phase 3 RCTs should be conducted only when there is some prior evidence to suggest a potential therapeutic benefit. If a cancer drug has failed to meet its primary end point in a phase 2 trial, there is neither therapeutic equipoise nor rationale to test it in a phase 3 RCT, and we question if it is against the ethical norms of participating in a phase 3 RCT. Institutional review boards and ethical committees should take a proactive step in discouraging the conduct of such phase 3 RCTs of cancer drugs that have already failed in phase 2 trials. Indeed, in a recent editorial, the US Food and Drug Administration questions the practice of conducting phase 3 RCTs of cancer drugs known to have poor activity in phase 2 trials.^76^

### Limitations

We included only those negative phase 3 RCTs published in journals with high impact factors; thus, our findings should be considered conservative. Indeed, our results reflected the best-case scenarios of the negative phase 3 RCTs, and the results would likely be worse for the negative phase 3 RCTs published elsewhere or not published at all. However, our search for phase 2 trials was conducted comprehensively without any journal or time restrictions. Another limitation of our study was the relative difficulty in accessing information on purchase or licensure of a drug from small pharmaceutical companies. Lack of a control group of positive phase 3 RCTs was another limitation; however, our objectives were to assess the association of negative phase 3 RCTs with sponsorship as well as to assess the association of negative phase 3 RCTs with patient survival, and therefore for our purpose, the cohort of negative trials alone sufficed. Furthermore, even though the trials were categorized into groups based on sponsorship, most trials will involve both industry and academia collaboration. We also acknowledge that in some instances of extraordinary responses in phase 1, it may be more efficient to take a drug directly to a phase 3 RCT; however, the activity and criteria for success (taking to a phase 3 RCT) must be prospectively defined in such early-phase trials.

## Conclusions

In this study of trials published in 2016 through 2018, approximately 40% of negative phase 3 RCTs in oncology were conducted without supporting phase 2 trials, and such phase 3 trials were sponsored by both academia and industry. On the basis of our results, proactive steps from regulators and ethical committees should be contemplated to encourage greater consideration before allowing conduct of phase 3 RCTs despite negative results from phase 2 trials in the interest of protecting patients and trial resources.

## Notes

Supplement. **eTable.** The 67 RCTs Included in the Study
