# Supplementary material for: Association of Industry and Academic Sponsorship With Negative Phase 3 Oncology Trials and Reported Outcomes on Participant Survival: A Pooled Analysis
Source: JAMA Netw Open. 2019 May 10;2(5):e193684. doi: 10.1001/jamanetworkopen.2019.3684 (PMC6512293; doi:10.1001/jamanetworkopen.2019.3684)
Supplement: Supplement. — eTable. The 67 RCTs Included in the Study [file jamanetwopen-2-e193684-s001.pdf]

Supplementary Online Content

Addeo A, Weiss GJ, Gyawali B. Association of industry and academic sponsorship with negative phase 3 oncology trials and reported outcomes on participant survival: a pooled analysis. *JAMA Netw Open*. 2019;2(5):e193684. doi:10.1001/jamanetworkopen.2019.3684

eTable. The 67 RCTs Included in the Study

This supplementary material has been provided by the authors to give readers additional information about their work.

© 2019 Addeo A et al. *JAMA Network Open*.

eTable. The 67 RCTs Included in the Study

Study name	Primary endpoint	OS Hazard ratio	95% confidence interval (lower limit/ upper limit)		N of patients		Sponsorship
ETNA	pCRrate				695	https://www.ncbi.nlm.nih.gov/pubmed/29327055	academia
NCT00820248	2 Y PFS	0.89	0.54	1.48	320	https://jamanetwork.com/journals/jamaoncology/fullarticle/2591160	industry
Metgastric	OS	0.82	0.59	1.15	562	https://www.ncbi.nlm.nih.gov/pubmed/27918764	industry
Ariser	OS and DFS	0.99	0.74	1.32	864	https://www.ncbi.nlm.nih.gov/pubmed/27787547	industry
RTOG0436	2 y OS	0.9	0.7	1.16	328	https://www.ncbi.nlm.nih.gov/pubmed/28687830	academia
NCT026850	OS	0.88	0.77	1.01	1074	https://www.ncbi.nlm.nih.gov/pubmed/28632865	academia
SELECT 1	PFS	1.05	0.85	1.3	505	https://www.ncbi.nlm.nih.gov/pubmed/28492898	industry
ImVigor211	OS	0.87	0.63	1.21	931	https://www.thelancet.com/journals/lancet/article/PIIS0140-6736(17)33297-X/fulltext	industry
SwogS9921	OS	1.06	0.79	1.43	961	https://www.ncbi.nlm.nih.gov/pubmed/29624463	academia
TROG 05.01	DFS	0.95	0.58	1.57	321	http://ascopubs.org/doi/pdfdirect/10.1200/JCO.2017.77.0941	academia
SUMIT	PFS	0.75	0.39	1.46	129	https://www.ncbi.nlm.nih.gov/pubmed/29528792	industry

© 2019 Addeo A et al. *JAMA Network Open*.

Noronha et al.	Local regional control	1.14	0.79	1.65	300	https://www.ncbi.nlm.nih.gov/pubmed/29220295	academia
PRODIGE 9	Tumor control duration	1.05	0.86	1.28	491	http://ascopubs.org/doi/abs/10.1200/JCO.2016.34.15_suppl.3531	academia
CHECKMATE 037	OS	0.95	0.73	1.24	272	http://ascopubs.org/doi/abs/10.1200/JCO.2016.71.8023	industry
IMPRESS	OS	1.44	1.07	1.94	265	http://ascopubs.org/doi/abs/10.1200/JCO.2017.73.9250	industry
Govindan et al.	OS	0.91	0.77	1.07	956	https://www.ncbi.nlm.nih.gov/pubmed/?term=28854067	industry
CONKO 005	DFS				436	https://www.ncbi.nlm.nih.gov/pubmed/28817370	academia
FIRSTANA	OS	1.01	0.85	1.2	1168	https://www.ncbi.nlm.nih.gov/pubmed/28753384	industry
Lee et al.	CR				299	https://www.ncbi.nlm.nih.gov/pubmed/28632487	academia
MATISSE	OS	1.3	0.95	1.78	188	http://ascopubs.org/doi/abs/10.1200/JCO.2016.71.7454	industry
DBCG 07-READ	5 y DFS	1.15	0.83	1.59	2012	https://www.ncbi.nlm.nih.gov/pubmed/28661759	academia
HELIOS	PFS	0.62	0.39	1.02	578	https://www.ncbi.nlm.nih.gov/pubmed/26655421	industry

© 2019 Addeo A et al. *JAMA Network Open*.

ALLIANCE	PFS	1.12	0.84	1.51	312	http://ascopubs.org/doi/full/10.1200/JCO.2016.71.3743	academia
SWOG S0518	PFS	1.16	0.88	1.55	427	https://www.ncbi.nlm.nih.gov/pubmed/28384065	academia
The GOIRC-AIFA FARM6PMFJM Trial	OS	0.78	0.58	1.06	204	https://www.ncbi.nlm.nih.gov/pubmed/28135143	academia
FACE TRIAL	5 y DFS	0.98	0.82	1.17	4136	https://www.ncbi.nlm.nih.gov/pubmed/28113032	industry
E1697	DFS rate	1.08	0.82	1.41	1150	http://ascopubs.org/doi/abs/10.1200/JCO.2016.70.2951	academia
ORCHARRD	2 y PFS	0.9	0.7	1.15	447	http://ascopubs.org/doi/abs/10.1200/JCO.2016.69.0198	industry
Met-Lung	OS	1.27	0.98	1.65	499	http://ascopubs.org/doi/abs/10.1200/JCO.2016.69.2160	industry
Beer et al.	OS	1.11	0.88	1.39	602	https://www.ncbi.nlm.nih.gov/pubmed/?term=28034081	industry
Powles et al.	PFS	0.96	0.7	1.31	466	https://www.ncbi.nlm.nih.gov/pubmed/?term=28034079	academia
NGR015	OS	0.94	0.75	1.18	400	https://www.thelancet.com/journals/lanonc/article/PIIS1470-2045(18)30193-1/abstract	industry
CRITICS	OS	1.01	0.84	1.22	788	https://www.thelancet.com/journals/lanonc/article/PIIS1470-2045(18)30132-3/fulltext	academia
METIV-HCC	OS	0.97	0.75	1.25	340	https://www.ncbi.nlm.nih.gov/pubmed/29625879	industry
FATA-GIM3	5 y DFS				3697	https://www.ncbi.nlm.nih.gov/pubmed/29482983	academia
BRIGHTNESS	% CR				634	https://www.ncbi.nlm.nih.gov/pubmed/29501363	industry
BRIM 8	DFS	0.76	0.49	1.18	498	https://www.ncbi.nlm.nih.gov/pubmed/29477665	industry

© 2019 Addeo A et al. *JAMA Network Open*.

PORTEC 3	OS and Failure FS (FFS)	0.76	0.54	1.06	686	https://www.thelancet.com/journals/lanonc/article/PIIS1470-2045(18)30079-2/fulltext	academia
SWOG S0819	PFS- OS	0.93	0.83	1.04	1313	https://www.ncbi.nlm.nih.gov/pubmed/29169877	academia
E1505	OS	0.99	0.82	1.19	1501	https://www.thelancet.com/journals/lanonc/article/PIIS1470-2045(17)30691-5/abstract	academia
SARAH	OS	1.15	0.94	1.41	467	https://www.ncbi.nlm.nih.gov/pubmed/29107679	industry
GOLD	OS	0.79	0.63	1	643	https://www.ncbi.nlm.nih.gov/pubmed/29103871	industry
RILOMET 1	OS	1.34	1.1	1.63	609	https://www.ncbi.nlm.nih.gov/pubmed/28958504	industry
DATA	DFS				1912	https://www.thelancet.com/journals/lanonc/article/PIIS1470-2045(17)30600-9/fulltext	industry
AFFINITY	OS	0.95	0.8	1.12	635	https://www.thelancet.com/journals/lanonc/article/PIIS1470-2045(17)30605-8/fulltext	industry
ACT IV	OS	1.01	0.79	1.3	745	https://www.ncbi.nlm.nih.gov/pubmed/28844499	industry
UK MRC OE05	OS	0.9	0.77	1.05	897	https://www.ncbi.nlm.nih.gov/pubmed/28784312	academia
TH CR-406/SARC021	OS	1.06	0.88	1.29	640	https://www.ncbi.nlm.nih.gov/pubmed/28651927	industry
CRUK/05/19	5 y time to tumor recurrence	1.04	0.88	1.21	4391	https://www.ncbi.nlm.nih.gov/pubmed/28600210	academia

© 2019 Addeo A et al. *JAMA Network Open*.

G-STS 1001	DFS 46 months rate	2.69	1.1	6.94	287	https://www.thelancet.com/journals/lanonc/article/PIIS1470-2045(17)30334-0/abstract	academia
SYNERGY	OS	0.93	0.79	1.1	1022	https://www.thelancet.com/journals/lanonc/article/PIIS1470-2045(17)30168-7/fulltext	industry
Rini et al.	OS	1.34	0.96	1.86	339	https://www.thelancet.com/journals/lanonc/article/PIIS1470-2045(16)30408-9/abstract	industry
Kerr et al.	3 years DFS	1.11	0.9	1.36	1941	https://www.thelancet.com/journals/lanonc/article/PIIS1470-2045(16)30172-3/fulltext	industry
Piperno-Neumann et al.	Event FS				318	https://www.thelancet.com/journals/lanonc/article/PIIS1470-2045(16)30096-1/fulltext	academia
Eichhorst et al.	PFS	1.034	0.62	1.724	561	https://www.thelancet.com/journals/lanonc/article/PIIS1470-2045(16)30051-1/fulltext	academia
Vansteenkiste et al.	DFS	1.04	0.86	1.24	2312	https://www.ncbi.nlm.nih.gov/pubmed/27132212	industry
Hecht et al.	OS	0.91	0.73	1.12	545	https://www.ncbi.nlm.nih.gov/pubmed/26628478	industry
Piccart-Gebhart et al.	DFS	0.80 and 0.91	0.62 and 0.71	1.03 and 1.16	8381	https://www.ncbi.nlm.nih.gov/pubmed/26598744	industry
Lipton et al.	molecular response at 12 months				307	https://www.ncbi.nlm.nih.gov/pubmed/27083332	industry

© 2019 Addeo A et al. *JAMA Network Open*.

Garcia-Manero et al.	OS	0.87	0.67	1.14	299	https://www.thelancet.com/journals/lanonc/article/PIIS1470-2045(16)00009-7/fulltext	industry
Reck et al.	OS	0.94	0.81	1.09	1132	http://ascopubs.org/doi/full/10.1200/JCO.2016.67.6601	industry
Ryan et al.	PFS	1.05	0.79	1.39	447	http://ascopubs.org/doi/full/10.1200/JCO.2016.67.6684	industry
Kelly et al.	DFS	1.13	0.88	1.45	973	http://ascopubs.org/doi/full/10.1200/JCO.2015.61.8918	industry
Smith et al.	OS	0.9	0.76	1.06	1028	http://ascopubs.org/doi/full/10.1200/JCO.2015.65.5597	industry
Crump et al.	DFS 3 years	1.04	0.74	1.47	758	http://ascopubs.org/doi/full/10.1200/JCO.2015.65.7171	industry
Kurzeder et al.	PFS	0.84	0.53	1.32	156	http://ascopubs.org/doi/full/10.1200/JCO.2015.66.0787	industry
Vergote et al.	PFS	0.99 for Far 1.25mg and 0.88 for far 2.5	0.78 and 0.68	1.27 and 1.13	1100	http://ascopubs.org/doi/abs/10.1200/jco.2015.63.2596	industry

OS= Overall survival, PFS = progression-free survival, DFS = Disease-free survival, pCR = pathological complete response rates

© 2019 Addeo A et al. *JAMA Network Open*.
